# Analysis of Organic Volatile Flavor Compounds in Fermented Stinky Tofu Using SPME with Different Fiber Coatings

**DOI:** 10.3390/molecules17043708

**Published:** 2012-03-26

**Authors:** Yuping Liu, Zhiwei Miao, Wei Guan, Baoguo Sun

**Affiliations:** 1 School of Food and Chemical Engineering, Beijing Technology and Business University, Beijing 100048, China; 2 Beijing Key Laboratory of Flavor Chemistry, Beijing 100048, China

**Keywords:** fermented stinky tofu, analysis, organic volatile flavor compounds, solid phase microextration

## Abstract

The organic volatile flavor compounds in fermented stinky tofu (FST) were studied using SPME-GC/MS. A total of 39 volatile compounds were identified, including nine esters, seven alcohols, five alkenes, four sulfides, three heterocycles, three carboxylic acids, three ketones, two aldehydes, one phenol, one amine and one ether. These compounds were determined by MS, and conformed by comparison of the retention times of the separated constituents with those of authentic samples and by comparison of retention indexes (RIs) of separated constituents with the RIs reported in the literature. The predominant volatile compound in FST was indole, followed by dimethyl trisulfide, phenol, dimethyl disulfide and dimethyl tetrasulfide. In order to find a better extraction time, the extraction times was optimized for each type of SPME fiber; the results show that the best extraction time for Carboxen/PDMS is 60 min, for PDMS/DVB 30 min, for DVB/CAR/PDMS 60 min and for PDMS 75 min. Of the four fibers used in this work, Carboxen/PDMS is found to be the most suitable to extract the organic volatile flavor compounds in fermented stinky tofu.

## 1. Introduction

Stinky tofu, also called chao tofu, chaw tofu or gray sufu, is one of the traditional Chinese soybean foods. Like some varieties of cheese, stinky tofu has an unpleasant smell, but it tastes delicious, so stinky tofu is also called Chinese cheese. According to the process technology, there are two kinds of stinky tofu: unfermented stinky tofu (UST) and fermented stinky tofu (FST) [1]. FST is usually gray, so it is also called gray sufu. UST is made by soaking tofu cubes in special stinky brines for 4–6 h. The tofu cubes aren’t fermented before soaking. The stinky brine is prepared by letting various ingredients, such as amaranth leaves, bamboo shoots, winter melon, fish, shrimp, *etc.*, in the brine carry out a natural fermentation with production of a strong stinky odor [2]. UST is very popular and often homemade in southern China. It is usually cooked and consumed as snack by deep-fat frying.

The processing method of FST is more complicated. Firstly, tofu cubes are inoculated with *Actinomucor elegans* and fermented in the incubator until they are covered with fungous mycelia to become moldy tofu. The fungous mycelium on the surface of moldy tofu cubes is removed; then the moldy tofu cubes are pickled with salt for 5–7 days. Finally, the salt-cured moldy tofu cubes are dipped and aged in the brine for 3–6 months [3]. As an appetizer, FST is popular in northern China and sold in jars. 

UST and FST are made by different methods, and thus they have different odor characteristics. Reports have been published on the volatile flavor compounds of UST [4], volatile compounds in the brine [5], and diversity of lactic acid bacteria in brine [6]. Beijing FST is very famous in China, under names like Wangzhihe stinky tofu, Laocaicheng stinky tofu, *etc.*, with Wangzhihe stinky tofu being perhaps the most well-known, but there are very few reports about organic volatile flavor compounds in Beijing FST. Therefore, the objective of the present study is to provide information on organic volatile flavor compounds in fermented stinky tofu (FST) from Beijing and to find which components lead to the offensive odor of FST.

The usual methods for extracting volatile substances from foodstuff are steam distillation [7], continuous seam distillation-extraction (SDE) [8], gas purge-and-trap technique [9], direct solvent extraction and solvent-assisted flavour evaporation (DSE-SAFE) [10] and headspace solid-phase microextraction (SPME) [11]. SPME offers some advantages over the other methods, such as being easy to perform, solvent free, sensitive and selective, *etc.*, so the SPME method was adopted in this paper.

## 2. Results and Discussion

### 2.1. The Optimization of Extraction Time

In order to find better extraction time for the four SPME fibers, the organic volatile flavor compounds in FST 1 were extracted for five different time periods (15, 30, 45, 60 and 75 min, respectively) at 50 °C. The results are listed in Table 1. 

**Table 1:** The number of identified organic volatile flavor compounds in FST 1.

SPME fiber	15 min	30 min	45 min	60 min	75 min
Carboxen/PDMS (75 μm)	27	32	32	38	27
PDMS/DVB (65 μm)	24	26	20	25	23
DVB/CAR/PDMS (50/30 μm)	15	16	16	18	12
PDMS (100 μm)	13	10	12	12	15

The results show that 60 min is more suitable for Carboxen/PDMS among the five chosen extraction time periods. The number of identified constituents is 38. The reason is mainly that the organic volatile flavor compounds in FST 1 need about 60 min to reach adsorption and desorption equilibrium in Carboxen/PDMS. If the extraction lasts longer, the adsorbed constituents which are in low abundance will be replaced by higher content constituents, which results in a smaller number of identified constituents. The film thickness of PDMS/DVB is thinner than that of Carboxen/PDMS, so it takes less time to reach adsorption and desorption equilibrium for the organic volatile flavor compounds in PDMS/DVB. The results indicate that 30 min is more suitable for PDMS/DVB. The film of DVB/CAR/PDMS is made from three different kinds of materials, and its film thickness is near to that of Carboxen/PDMS. The more suitable extraction time period for DVB/CAR/PDMS is also 60 min. Among the five chosen extraction time periods, 75 min is better for PDMS. The reason is that the film of PDMS is the thickest of the four kinds of fibers, so the volatile organic flavor compounds need more time to reach adsorption and desorption equilibrium. 

**Figure 1:**
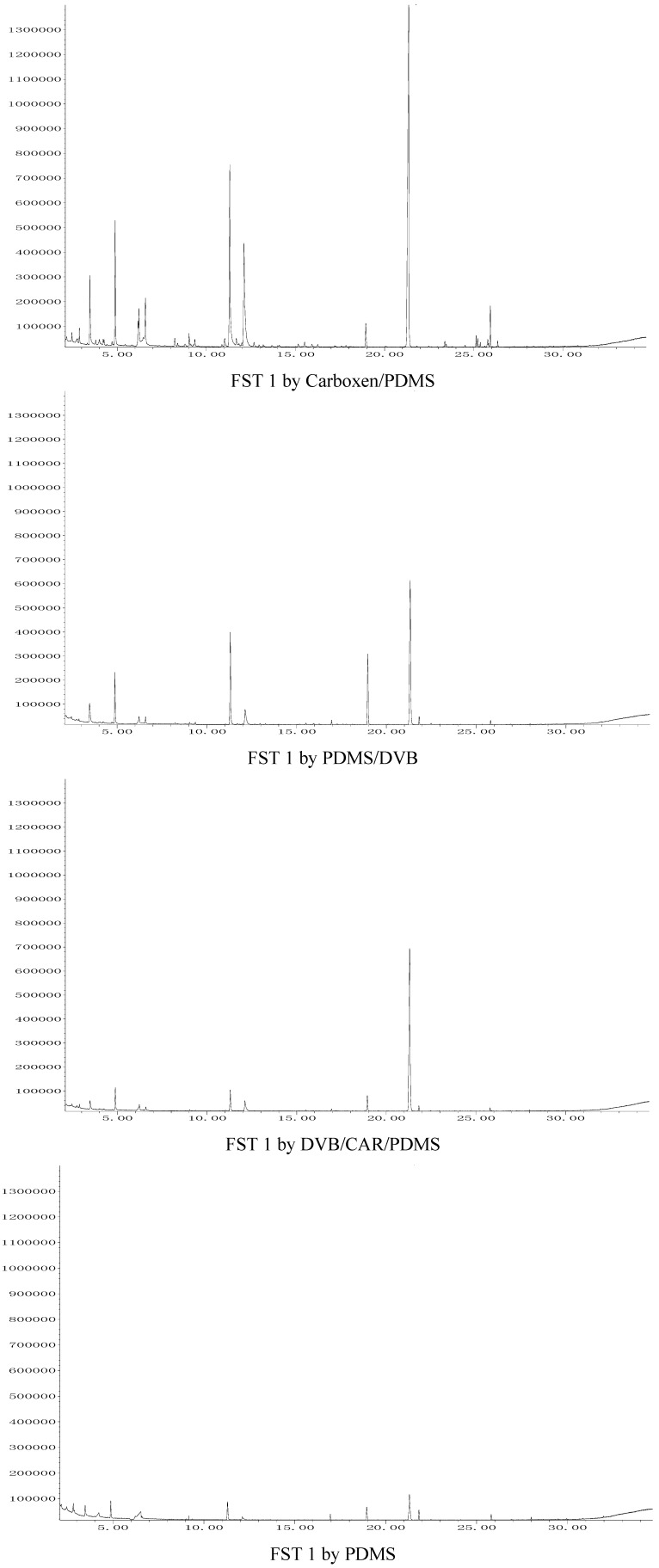
The total ion chromatograms of organic flavor compounds in FST 1 extracted with the four SPME fibers corresponding to Table 2.

**Figure 2:**
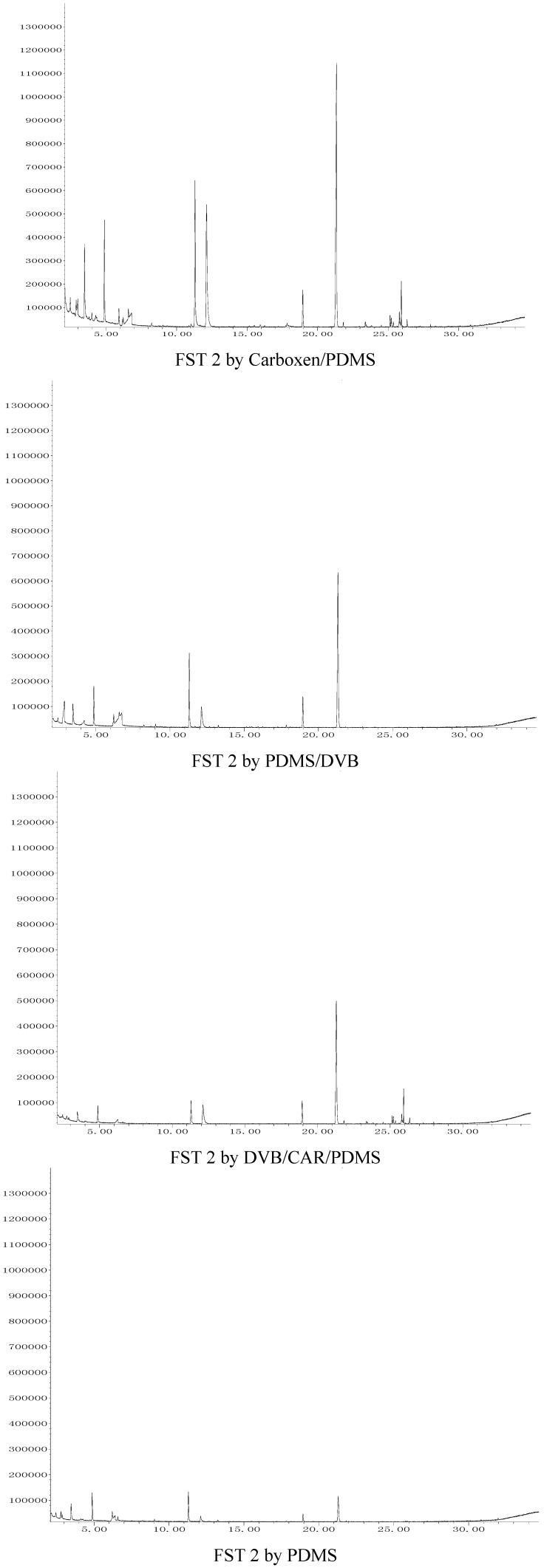
The total ion chromatograms of organic flavor compounds in FST 2 extracted with the four SPME fibers corresponding to Table 2.

The organic volatile flavor compounds in FST 2 were extracted for 60 min using Carboxen/PDMS, 30 min with PDMS/DVB, 60 min with DVB/CAR/PDMS and 75 min w PDMS, respectively. The total ion chromatograms of organic flavor compounds in FST 1 extracted with the four SPME fibers corresponding to Table 2 are shown in Figure 1, and the total ion chromatograms of organic flavor compounds in FST 2 extracted with the four SPME fibers corresponding to Table 2 are shown in Figure 2. The analytical results for FST 1 and FST 2 are listed in Table 2.

### 2.2. The Effect of SPME Fiber Coating on Analytical Results

From the point of view of the number of identified compounds, it is clear that the amount of identified compounds is the largest using Carboxen/PDMS for extracting volatile flavor constituents in FST1 and FST2. The result is agreement with Yu’s, who analyzed the volatile compounds in traditional smoke-cured bacon with different fiber coatings using SPME and found that Carboxen/PDMS showed the best results [11]. Maybe Carboxen/PDMS is the most suitable fiber for extracting volatile flavor compounds in food among the four fibres. 

The effect of PDMS/DVB is moderate. It can extract alcohol, phenol, carboxylic acids, ester, sulfide and heterocycle compounds. However, alkene compounds are not identified; the reasons are in two aspects. One is that the contents of alkenes are lower; the other is that PDMS/DVB may be not suitable for extracting alkene compounds. 

The effect of DVB/CAR/PDMS is poorer than that of PDMS/DVB, and organic acids are not identified. At the same time, the number of aldehyde and ketone compounds identified is also much less. 

Of the four fibers used in this work, when PDMS was used for extracting volatile compounds, the number of identified compounds was the least. Aldehyde and ketone compounds and alkene compounds are not identified; perhaps PDMS is not fit for extracting these three kinds of organic compounds. However, the three organic acids are all identified by using PDMS. 

From the point of view of the relative peak area of identified compounds, the extract efficiency of four fibers on alcohols and phenol in FST1 and FST2 is close. The sum of relative peak area of alcohols and phenol in FST2 is bigger than that in FST1. It is 12.82–24.94% in FST2, but it is 9.31–17.93% in FST1. The results showed that the content of alcohols and phenol in FST2 might be indeed higher than that in FST1. 

**Table 2:** Identification of organic volatile flavor compounds in FST1 and FST2 using SPME.

Volatiles	CAS#	RI/RI ^*a^	Qual ^b^	I method ^c^	Carboxen/PDMS		PDMS/DVB		DVB/CAR/PDMS		PDMS	
					Peak area (%)		Peak area (%)		Peak area (%)		Peak area (%)	
					FST1	FST2	FST1	FST2	FST1	FST2	FST1	FST2
					60 min	60 min	30 min	30 min	60 min	60 min	75 min	75 min
*Alcohols and phenol*												
1-Propanol	71-23-8	<604/568^[12]^	72	MS, S	0.49	0.98	0.19	0.62	0.86	0.98	3.41	2.48
1-Butanol	71-36-3	663/662^[13]^	91	MS,RI,S	3.92	5.03	4.55	4.18	2.81	3.86	7.06	9.84
3-Methyl-1-butanol	123-51-3	734/734^[12]^	72	MS,RI,S	0.17	ND	0.22	ND	0.10	ND	0.10	ND
1-Hexanol	111-27-3	871/871^[14]^	83	MS,RI,S	0.54	0.31	0.29	0.29	ND	ND	ND	0.64
1-Octen-3-ol	3391-86-4	983/983^[15]^	70	MS,RI	0.76	0.16	0.26	0.21	0.31	ND	ND	ND
Phenylethyl alcohol	60-12-8	1120/1121^[16]^	90	MS,RI,S	0.27	0.33	0.32	0.19	ND	ND	ND	ND
4-Methyl-1-(1-m ethylethyl)-3-Cyclohexen-1-ol	562-74-3	1183/1180^[17]^	95	MS,RI	0.10	0.54	ND	0.38	ND	ND	ND	ND
Phenol	108-95-2	996/995^[18]^	95	MS,RI,S	11.68	17.59	6.16	6.95	5.23	10.33	2.99	5.05
Total					17.93	24.94	11.99	12.82	9.31	15.17	13.56	18.01
*carboxylic acids*												
Acetic acid	64-19-7	628/637^[19]^	72	MS,RI,S	ND	1.89	ND	7.05	ND	ND	6.53	3.72
Propanoic acid	79-09-4	719/740^[20]^	91	MS,RI,S	0.14	0.21	0.24	1.87	ND	ND	6.37	1.09
Butanoic acid	107-92-6	823/822^[21]^	91	MS,RI,S	1.40	2.85	0.40	6.23	ND	ND	13.50	7.61
Total					1.54	3.95	0.64	15.15	0	0	26.40	12.42
*Ester*												
Ethyl acetate	141-78-6	617/612^[22]^	72	MS,RI,S	0.67	0.85	0.27	ND	0.72	0.48	ND	2.60
Ethyl propanoate	105-37-3	713/714^[12]^	72	MS,RI	0.26	ND	0.20	ND	0.21	ND	ND	ND
n-Propyl acetate	109-60-4	716/712^[12]^	72	MS,RI	0.29	0.60	0.19	ND	0.17	ND	ND	ND
Ethyl butanoate	105-54-4	803/803^[19]^	93	MS,RI,S	3.51	0.89	2.64	2.26	2.83	2.83	2.07	6.01
Butyl acetate	123-86-4	815/812^[12]^	83	MS,RI,S	3.52	3.10	1.32	7.52	0.80	0.59	2.18	2.96
3-Methyl-1-butyl acetate	123-92-2	877/877^[23]^	83	MS,RI,S	0.19	ND	0.15	ND	ND	ND	ND	ND
Propyl butanoate	105-66-8	899/900^[24]^	86	MS,RI	0.72	ND	0.26	0.37	0.39	ND	0.39	0.92
Butyl propanoate	590-01-2	909/910^[12]^	83	MS,RI	0.44	ND	0.47	0.11	0.15	ND	0.40	ND
Hexyl acetate	142-92-7	1014/1008^[24]^	90	MS,RI	0.36	ND	0.13	0.15	ND	ND	ND	ND
Total					9.96	5.44	5.63	10.41	5.27	3.90	5.04	12.49
*Aldehyde and ketone*												
Benzaldehyde	100-52-7	962/962^[25]^	97	MS,RI,S	0.73	0.26	0.47	ND	0.31	ND	ND	ND
benzeneacetaldehyde	122-78-1	1046/1046^[26]^	78	MS,RI,S	0.17	0.10	ND	ND	ND	ND	ND	ND
2-Pentanone	107-87-9	689/687^[27]^	70	MS,RI	0.31	0.23	0.13	ND	ND	ND	ND	ND
2-Heptanone	110-43-0	891/890^[28]^	83	MS,RI,S	0.21	ND	ND	ND	ND	ND	ND	ND
2-Methyl-2-cyclopenten-1-one	1120-73-6	907/914^[29]^	74	MS,RI	0.12	ND	ND	ND	ND	ND	ND	ND
Total					1.54	0.59	0.60	0	0.31	0	0	0
*Sulfide*												
Dimethyl disulfide	624-92-0	743/742^[30]^	98	MS,RI,S	6.52	6.10	7.98	5.32	5.20	3.99	6.92	12.36
Dimethyl trisulfide	3658-80-8	971/972^[22]^	94	MS,RI,S	14.55	12.62	17.12	11.93	5.83	6.51	9.44	16.88
Methyl (methylthio) methyl disulfide	42474-44-2	1129/1139^[31]^	86	MS,RI	0.19	0.11	0.13	0.21	ND	ND	ND	ND
Dimethyl tetrasulfide	5756-24-1	1221/1220^[32]^	93	MS,RI	1.53	2.83	13.16	5.03	4.10	5.84	6.69	4.16
Total					22.79	21.66	38.39	22.49	15.13	16.34	23.05	33.40
*Heterocycles*												
2-Pentylfuran	3777-69-3	992/992^[12]^	87	MS,RI	0.34	0.07	0.21	0.13	0.25	ND	ND	ND
2-Pentylthiophene	4861-58-9	1163/1164^[33]^	83	MS,RI	0.09	ND	0.17	ND	ND	ND	ND	ND
Indole	120-72-9	1304/1303^[34]^	97	MS,RI,S	36.76	31.01	38.64	37.38	64.79	45.55	19.78	21.00
Total					37.19	31.08	39.02	37.51	65.04	45.55	19.78	21.00
*Alkene*												
Limonene	138-86-3	1030/1030^[35]^	95	MS,RI	0.14	ND	ND	ND	ND	ND	ND	ND
Copaene	3856-25-5	1382/1382^[36]^	96	MS,RI	0.31	0.35	ND	ND	ND	ND	ND	ND
alpha-Caryophyllene	6753-98-6	1468	95	MS	0.49	0.62	ND	ND	ND	1.59	ND	ND
Aromadendrene	109119-91-7	1475/1470^[37]^	76	MS,RI	0.20	0.28	ND	ND	ND	0.55	ND	ND
alpha-Panasinsen	56633-28-4	1532	94	MS	0.29	0.44	ND	ND	ND	1.16	ND	ND
Total					1.43	1.69	0	0	0	3.30	0	0
*Others*												
Dimethylamine	124-40-3	<604	72	MS	0.17	ND	ND	ND	ND	ND	0.82	0.01
Eucalyptol	470-82-6	1033/1033^[38]^	98	MS,RI,S	0.05	ND	0.13	0.34	ND	ND	ND	0.61
Total					0.22	0	0.13	0.34	0	0	0.82	0.62
All total					92.60	89.35	96.40	98.72	95.06	84.26	88.65	97.94

^a ^RI: retention index, RI*: retention index from literature; ^b ^Qual: the similar degree of their mass spectra comparing with those contained in the Nist08 database; ^c ^Identification method: MS, compared with Nist 08 Mass Spectral Database; RI, agrees with retention index of literatures; S, agrees with mass spectrum of authentic standards; ND: not detected.

The extract efficiency of Carboxen/PDMS and PDMS/DVB on carboxylic acids is close; the sum of relative peak area of fatty acids in FST2 is larger than that in FST1. However, when PDMS was used, the sum of relative peak area of fatty acids in FST2 is smaller than that in FST1. We think there may be two reasons. One is that PDMS is the best fiber for extracting carboxylic acids; the other is that the value of peak area is relative. When more constituents are identified, the peak area of every constituent is smaller. Among the four fibers, the number of constituents identified in FST1 is the least by using PDMS.

The extraction efficiencies of Carboxen/PDMS and DVB/CAR/PDMS on esters and PDMS/DVB and PDMS on esters are close. When Carboxen/PDMS and DVB/CAR/PDMS were used, the peak area of esters in FST1 (9.96% and 5.27%) is bigger than that in FST2 (5.44% and 3.90%). However, when PDMS/DVB and PDMS were used, the peak area of esters in FST1 (5.63% and 5.04%) is smaller than that in FST2 (10.41% and 12.49%). The reason may be that the peak area is a relative value. It is affected by a number of factors, such as the kind of fiber, the thickness of fiber, the number of identified constituents, the polarity of constituents, *etc.*

The extraction efficiency of Carboxen/PDMS on aldehydes and ketone is best among the four fibers. The total peak areas in FST1 and FST2 were 1.54% and 0.59%, respectively. When PDMS/DVB and DVB/CAR/PDMS were used, aldehydes and ketone were identified only in FST1. When PDMS was used, aldehydes and ketone were not identified in FST1 and FST2. The results indicated that the total content of aldehydes and ketone was lower in FST, especially in FST2. 

The extraction efficiencies of Carboxen/PDMS and PDMS/DVB on sulfide and DVB/CAR/PDMS and PDMS on sulfide are close. When these fibers were used, the peak area of sulfide in FST1 (22.79% and 38.39%) is bigger than that in FST2 (21.66% and 22.49%). However, when DVB/CAR/PDMS and PDMS were used, the peak area of sulfide in FST1 (15.13% and 23.05%) is smaller than that in FST2 (16.34% and 33.40%). The main reason may be that the peak area is a relative value, too. 

The relative peak areas of indole in FST1 and FST2 were close when using Carboxen/PDMS, PDMS/DVB and PDMS, but the value by PDMS was smaller than those by Carboxen/PDMS and PDMS/DVB. Maybe PDMS was better for extracting fatty acids, and this made the relative peak areas of indole become smaller. Among the four fibers, the relative peak areas of indole in FST1 (64.79%) and FST2(45.55%) were biggest by using DVB/CAR/PDMS. Maybe the reason was that indole was adsorbed easier by DVB/CAR/PDMS and the total number of identified compounds was less.

### 2.3. Volatile Compounds of FST

From Table 2, it could be seen that total of 39 volatile compounds are identified in FST samples, including nine esters, seven alcohols, five alkenes, four sulfides, three heterocycles, three carboxylic acids, three ketones, two aldehydes, one phenol, one amine and one ether (eucalyptol). The predominant volatile compound in FST is indole, followed by dimethyl trisulfide, phenol, dimethyl disulfide and dimethyl tetrasulfide. 

The number of ester compounds is the largest among the detected compounds. The esters identified are acetate, propanoate and butanoate. They are formed by the esterification reaction of organic acids with alcohols under catalysis of enzymes, which were produced by molds. The three kinds of organic acids and most of alcohols forming these esters are also identified in the FST sample. These ester compounds can impart FST with fruity notes and make the odor of FST lifting and diffusive. Ethyl acetate has pleasant ethereal-fruity like aroma; 3-methyl-1-butyl acetate has sweet, banana, fruity with a ripe ester nuance; hexyl acetate has green, fruity, sweet, fatty odor; ethyl propanoate has fruity, rum, fermented and pineapple aroma; ethyl butanoate has sweet, fruity and tutti frutti odor [39]. Carboxen/PDMS and PDMS/DVB are more suitable for the extraction of ester compounds. When they were used, nine esters were all identified.

Alcohol compounds are also isolated from FST, and their formations may be due to the fermentation of carbohydrates from soybean during the ripening step. 1-Butanol is the main linear aliphatic alcohol. The detected alkenes, mainly including limonene, copaene, α-caryophyllene, aromadendrene and α-panasinsen, may be from materials used in the manufacture process of stinky tofu, like pepper, which contains these alkene compounds [40]. Carboxen/PDMS and PDMS/DVB are also suitable for the extraction of alcohol compounds

Phenol is the only phenol compound identified in FST. Maybe it comes from the decomposition of tyrosine. The reason is that structural formula of tyrosine contains the structure of phenol, and the content of tyrosine fluctuates and even sometimes cannot be detected during the ripening process [41]. Phenol is not only a flavor compound but also a kind of bactericide. As a flavor compound, it has phenolic, plastic and rubber odor [39]; as a kind of bactericide, it can extend the shelf life of stinky tofu.

The detected organic acids are acetic acid, propanoic acid and butanoic acid. They are considered from the hydrolysis of soybean lipids [3] and from the action of deaminase of amino acids. In the course of deaminase, ammonia compounds are also formed. The isolation of dimethylamine in the experiments can prove this statement. PDMS was more suitable for the extraction of carboxylic acids. When it was used, three carboxylic acids were all identified in FST 1 and FST 2.

Aldehydes and ketones might be formed by beta-oxidation of fatty acids, which generated a few of important flavor compounds [11]. Two aromatic aldehydes, two methylketones and a cyclopentenone are observed in FST. Benzaldehyde is described as almond-like aroma and can give FST a nutty aroma, benzeneacetaldehyde is described as floral, sweet, sauce and soy sauce odor and can impart FST savory odor, 2-pentanone and 2-heptanone have sweet, fruity and ethereal aroma and make FST have sweet nuance, and 2-methyl-2-cyclopenten-1-one gives FST wood smoke notes. Among the four fiber used, Carboxen/PDMS is the most suitable for the extraction of these compounds

Furans and their derivatives are considered derived from Maillard reactions [11]. 2-Pentylfuran and 2-pentylthiophene are detected in FST. Furan derivatives possess caramel, sweet, roasted, burnt and sugar notes.

Four sulfides, including dimethyl disulfide, dimethyl trisulfide, dimethyl tetrasulfide and methyl (methylthio) methyl disulfide, are isolated from FST. They arise from the degradation of amino acids containing sulfur. Stinky tofu is made of soybean which is rich in proteins; the protein in the tofu is hydrolyzed by the microbial proteases to form amino acids, among which cysteine and methionine are sulfur-containing amino acids. During the ripening process of stinky tofu, the contents of the two sulfur-containing amino acids change, especially the content of methionine. When the ripening time is 80 day, methionine is not detected [42]. Dimethyl disulfide is with sulfurous, cabbage and onion odor; dimethyl trisulfide has sulfureous, alliaceous, cooked, savory, meaty, eggy and onion note; dimethyl tetrasulfide is described as sulfureous, galic and meaty odor; methyl (methylthio) methyl disulfide has strong sulfureous and onion odor [43]. Although most of sulfides are identified by using the four fibers, the number of identified compounds is more with Carboxen/PDMS and PDMS/DVB.

Indole is identified in FST and its content is the highest in the volatile flavor constituents of FST. It might rise from the degration of tryptophan. The reason is that the structure of tryptophan contains the structure of indole, and the content of tryptophan decreases during the ripening process [42]. Indole has animal and fecal odor [39], it gives FST an unpleasant odor. 

From Table 2, it can be seen that FST 1 have more volatile flavor compounds than FST 2. Maybe there are some differences between their manufacturing processes. Esters, alcohols, aldehydes and ketones can give FST fruity and sweet odors, but the aroma intensities of indole and sulfides exceed their aroma intensities. The reason is that indole and four sulfides all have very low odor threshold values and relatively higher contents. Indole and four sulfides are characteristic volatile flavor constituents of FST and they give FST its very strong unpleasant odor.

## 3. Materials and Methods

### 3.1. Materials

FST 1 was purchased from Beijing Wangzhihe Food Group Co., LTD, which is the most famous manufacturer of FST with more than 300 years history in China. FST 2 was obtained from Beijing Laocaichen Food Co., LTD. Manufacturing day of FST 1 is 20110703, and that of FST 2 is 20110618. In order to keep the sample uniform, FST was mashed into a slurry with a glass rod before the experiments. 

Four kinds of SPME fibers with different coats were purchased from Supelco Inc. (Bellefonte, PA, USA). They are carboxen/polydimethylsiloxane (Carboxen/PDMS, 75 μm thickness, black color), polydimethylsiloxane/divinylbenzene (PDMS/DVB, 65 μm thickness, blue color), divinylbenzene/carboxen/polydimethylsiloxane (DVB/CAR/PDMS, 50/30 μm thickness, gray color), polydimethylsiloxane (PDMS, 100 μm thickness, red color). The fibers used were preconditioned prior to the analysis in the injection port of the gas-chromatograph according to the instructions suggested by the manufacturer.

C_6_-C_23_ normal alkanes for calculating the retention indices (RI) were purchased from Aldrich Chemical Co. Authentic reference aroma compounds were obtained from Beijing Peking University Zoteq Co., LTD.

### 3.2. Headspace Solid-Phase Microextraction-Gas-Chromatography-Mass Spectrometry (Headspace-SPME-GC-MS)

#### 3.2.1. SPME Sampling

FST slurry (20 g) and a magnetic stir bar were placed in a 50 mL vial (special for SPME). Before the SPME fiber was inserted into the vial, the vial was sealed with one Teflon cover and equilibrated for 20 min in a 50 °C water bath. After that, the fiber was exposed in the upper space of the sealed vial to extract compounds for some time. 

#### 3.2.2. Analysis by GC-MS

An Agilent 6890 GC coupled with a 5973i mass spectrometer (Agilent Technologies, Palo Alto, CA, USA) was used. The GC was equipped with a HP-5MS capillary column (30 m × 250 μm i.d. × 0.25 μm, Agilent Technologies). Helium was the carrier gas with a constant flow of 1 mL/min to the column. The initial oven temperature was at 40 °C, holding for 2 min, then raised to 150 °C at 5°C/min; and finally raised to 280 °C at 15 °C/min, holding for 2 min. The injection port was in splitless mode. The mass detector was operated at 150 °C in electron impact mode at 70 eV. The ion source temperature was at 230 °C and the transfer line temperature was at 250 °C. The chromatograms were recorded by monitoring the total ion currents in the 15–450 mass range. MS was detected with 2 min solvent delay. Analysis of the sample at each condition was repeated 1 times. C6-C23 *n*-alkanes were run under the same chromatographic conditions in order to calculate the retention indices (RI) of detected compounds. Compounds were identified by comparing their mass spectra with those contained in the NIST08 database, and confirmed by comparison of the retention times of the separated constituents with those of the authentic samples and by comparison of retention indexes (RIs) of the separated constituents with the RIs reported in the literature.

## 4. Conclusions

The objectives of the present investigation were to analyze the profile of the organic volatile flavor compounds in fermented stinky tofu using SPME, to optimize the extraction time of four kinds of fiber and to study the effect of fiber coating on the volatile profile of FST. The results shows that: (1) a total of 39 volatile compounds are identified in FST samples, including nine esters, seven alcohols, five alkenes, four sulfides, three heterocycles, three carboxylic acids, three ketones, two aldehydes, one phenol, one amine and one ether (eucalyptol). The predominant volatile compound in FST is indole, followed by dimethyl trisulfide, phenol, dimethyl disulfide and dimethyl tetrasulfide; (2) the best extraction times for Carboxen/PDMS, PDMS/DVB, DVB/CAR/PDMS and PDMS are 60, 30, 60 and 75 min, respectively; (3) of the four fibers used in this work, Carboxen/PDMS fiber is found to be the most suitable to extract the organic volatile flavor compounds in fermented stinky tofu; (4) indole and four sulfides are characteristic volatile flavor constituents of FST and they give FST its offensive odor.
